# Effect of the Environmental Stimuli upon the Human Body in Winter Outdoor Thermal Environment

**DOI:** 10.1155/2013/418742

**Published:** 2013-06-19

**Authors:** Yoshihito Kurazumi, Emi Kondo, Jin Ishii, Tomonori Sakoi, Kenta Fukagawa, Zhecho Dimitrov Bolashikov, Tadahiro Tsuchikawa, Naoki Matsubara, Tetsumi Horikoshi

**Affiliations:** ^1^School of Life Studies, Sugiyama Jogakuen University, 17-3 Hoshigaoka-motomachi, Chikusa-ku, Nagoya, Aichi 464-8662, Japan; ^2^Nagoya Institute of Technology, Gokiso-cho, Showa-ku, Nagoya, Aichi 468555, Japan; ^3^Faculty of Education, Gifu University, 1-1 Yanagido, Gifu 501-1193, Japan; ^4^International Young Researchers Empowerment Center, Shinshu University, 3-15-1 Tokida, Ueda, Nagano 386-8567, Japan; ^5^Department of Architecture, Kyushu Sangyo University, 2-3-1 Matsukadai, Higashi, Fukuoka 813-8503, Japan; ^6^International Centre for Indoor Environment and Energy, Technical University of Denmark, Nils Koppels Allé, Building 402, 2800 Kongens Lyngby, Denmark; ^7^School of Human Science & Environment, University of Hyogo, 1-1-12 Hon-cho, Shinzaike, Himeji, Hyogo 670-0092, Japan; ^8^Division of Environmental Sciences, Graduate School of Kyoto Prefectural University, Nakaragi-cho, Shimogamo, Sakyo-ku, Kyoto 606-8522, Japan; ^9^Department of Techno-Business Administration, Graduate School of Nagoya Institute of Technology, Gokiso-cho, Showa-ku, Nagoya, Aichi 466-8555, Japan

## Abstract

In order to manage the outdoor thermal environment with regard to human health and the environmental impact of waste heat, quantitative evaluations are indispensable. It is necessary to use a thermal environment evaluation index. The purpose of this paper is to clarify the relationship between the psychological thermal responses of the human body and winter outdoor thermal environment variables. Subjective experiments were conducted in the winter outdoor environment. Environmental factors and human psychological responses were measured. The relationship between the psychological thermal responses of the human body and the outdoor thermal environment index ETFe (enhanced conduction-corrected modified effective temperature) in winter was shown. The variables which influence the thermal sensation vote of the human body are air temperature, long-wave thermal radiation and short-wave solar radiation. The variables that influence the thermal comfort vote of the human body are air temperature, humidity, short-wave solar radiation, long-wave thermal radiation, and heat conduction. Short-wave solar radiation, and heat conduction are among the winter outdoor thermal environment variables that affect psychological responses to heat. The use of thermal environment evaluation indices that comprise short-wave solar radiation and heat conduction in winter outdoor spaces is a valid approach.

## 1. Introduction 

Much research relating to the control of air conditioning systems for living environments and office spaces has been performed but that on outdoor spaces is incomplete. It has been shown that, in addition to physical and physiological environmental factors, psychological environmental stimuli are also important for the determination of thermal comfort. In contrast to indoor spaces, it is not just the thermal environment stimuli but also the environmental complex consisting of visual and auditory stimuli that has an influence on comfort. Adaptation to the thermal environment according to cultural background, experience of being in hot/cold thermal environments and the like, sense of expectation of the thermal environment, behavioural thermal regulation, and the effect of thermal environment history such as the time spent exposed to it are strongly apparent [1–8]. Although thermal comfort is the subject of research, it is treated as nonspecific and comprehensive rather than particular [9]. In the case of restricting the responses to thermal stimuli in instructions for the experiment and not having subjects respond on thermal sense, a nonspecific evaluation is a thermal evaluation but is also the evaluation of a comprehensive impression of space by environmental stimuli other than thermal stimuli, such as visual or auditory stimuli [10–14]. In contrast, a specific evaluation is when the test subjects are made to focus on and evaluate thermal stimuli alone as instructed by the research staff. Research specifically on thermal factors is rare in comparison with research that deals with a thermally nonspecific comprehensive sense of comfort [15–19]. Such research is carried out with the objective of finding the comfort zone of an outdoor space. 

The hot and cold that humans sense is typically investigated by means of air temperature. In a summer outdoor space, however, strong solar radiation from the sun gives a hot feeling, a strong wind gives a cool feeling, high humidity gives a muggy feeling, and a heated road surface feels so hot that it cannot be touched. Accordingly, it is necessary to include not only air temperature but also the environmental elements of thermal radiation, convection, humidity, and heat conduction in assessment of the thermal sense of humans. That is to say, it is necessary to make an explicit relationship between the thermal sense of the human body and the thermal environment evaluation indices that support the heat balance of the human body. 

Givoni et al. [20], Oliveira and Andrade [21], Eliasson et al. [22], Nikolopoulou et al. [3], Nikolopoulou and Lykoudis [7], Ishii et al. [23], and others show the relationship between the physical environmental factors of the outdoor space and the thermal sensation of a person and demonstrate that the outdoor environmental factors that influence the thermal sensation vote are air temperature, air velocity, and solar radiation. No research, however, considers the factor of heat conduction. Kurazumi et al. [18] clarify the effect that the environmental factors of a summer outdoor space have on sensation and physiological temperature. They demonstrate that the summer outdoor environmental factors that influence the thermal sensation of the human body are heat conduction, humidity, and short-wave solar radiation. They also demonstrate that the factors that affect the thermal comfort of the human body are air velocity, heat conduction, and humidity. In outdoor spaces, solar radiation from the sun and air velocity become strong, and the influence of sensational and physiological temperature increases. In addition, long-wave thermal radiation from the local objects on the ground surface forms a heterogeneous thermal radiation environment because of the effect of direct solar radiation, and the wind direction and current speed form a transient state that is constantly fluctuating. Accordingly, the effect on the human body of the physical environmental factors that compose sensational and physiological temperature is remarkably large in comparison with indoor space. 

Enhanced conduction-corrected modified effective temperature (ETFe) [24] and universal effective temperature (ETU) [25] are thermal environment evaluation indices that examine outdoor space and take solar radiation and heat conduction as environmental factors. ETFe and ETU can temperature-convert the effect of air temperature and air velocity, long-wave thermal radiation in outdoor space, short-wave solar radiation, contact member's surface temperature, and humidity into individual meteorological elements. The addition of each temperature-such converted factor is also possible, and quantifying the composite effect on sensation in the outdoor space as well as the discrete effect of each meteorological element is possible on the same evaluation axis. Verification tests that demonstrate the relationship of the physiological and psychological effects on the human body have been carried out in summer for ETFe, and its suitability as an outdoor environmental evaluation index has been demonstrated [18]. Its correlation with outdoor thermal environmental factors in winter has not, however, been investigated. 

Accordingly, this research shows the relationship between the outdoor thermal environmental evaluation index for winter and the psychological response of the human body by using ETFe to temperature-convert the effect of air temperature and air velocity, long-wave thermal radiation in outdoor space, short-wave solar radiation, contact member's surface temperature, and humidity and arrange them on the same evaluation axis. It goes on to investigate the individual environmental factors that should be incorporated as evaluation factors for an outdoor thermal environment. 

## 2. Experiment Design

### 2.1. ETFe

The thermal environment evaluation index for an outdoor space ETFe was developed by Kurazumi et al. [24]. It was designed to include the influence of solar radiation and the concept of conduction-modified corrected operative temperature (ETF) [26]. ETFe can temperature-convert the effect of air velocity and difference in attitude, long-wave thermal radiation in outdoor space, short-wave solar radiation, contact part's surface temperature, and humidity into individual meteorological elements. 

The effect of these five environmental factors on the heat balance of the human body can be expressed by a newly defined thermal environment evaluation index as follows: convective heat transfer area combined thermal velocity field for air velocity (TVF_hta_); radiant heat transfer area combined effective radiation field concerning long-wave thermal radiation in an outdoor space for long-wave thermal radiation in an outdoor space (ERF_htaL_); radiant heat transfer area combined effective radiation field concerning short-wave solar radiation in an outdoor space for solar radiation (ERF_htaS_); conductive heat transfer area combined effective conductive field for the contact member's surface temperature (ECF_hta_); and effective humid field in enhanced conduction-corrected modified effective temperature in humidity ETFe for humidity (EHF_ETFe_). The addition of each temperature-converted factor is also possible, and quantifying the composite effect on sensation in the outdoor space as well as the discrete effect of each meteorological element is possible on the same evaluation axis.

Consider

(1) ETFe=Ta+TVFhtahfL+ERFhtaLhfL+ECFhtahfL +EHFETFehfL+ERFhtaShfL,

(2) $$\begin{array}{c} \text{TV}{\text{F}}_{\text{hta}}=\left(h_{o}\text{fcl}\text{Fclo}f_{\text{conv}}-h_{c}\text{fcl}\;\text{Fcl}\;f_{\text{conv}}\right)\left(t_{s}-t_{a}\right), \end{array}$$

(3) $$\begin{array}{c} \text{ER}{\text{F}}_{\text{htaL}}=h_{\text{rL}}\text{fcl}\;\text{Fcl}\;f_{\text{rad}}\left(t_{\text{rL}}-t_{a}\right), \end{array}$$

(4) $$\begin{array}{c} \text{EC}{\text{F}}_{\text{hta}}=h_{d}\text{Fcld}\;{\text{f}}_{\text{cond}}\left(t_{f}-t_{a}\right), \end{array}$$

(5) $$\begin{array}{c} \text{EH}{\text{F}}_{\text{ETFe}}=Lwh_{c}\text{fcl}\;\text{Fpcl}\left(p_{a}-0.5p_{\text{ETFe}}^{*}\right), \end{array}$$

(6) $$\begin{array}{c} \text{ER}{\text{F}}_{\text{htaS}}=R_{s}, \end{array}$$

(7) $$\begin{array}{c} h_{\text{fL}}=h_{o}\text{fcl}\;\text{Fclo}\;f_{\text{conv}}+h_{\text{rL}}\text{fcl}\;\text{Fcl}\;f_{\text{rad}}+h_{d}\text{Fcld}\;f_{\text{cond}}, \end{array}$$

where ETFe is the enhanced conduction-corrected modified effective temperature [K]; *T*_*a*_ is the air temperature [K];  TVF_hta_ is the convective heat transfer area of the combined thermal velocity field [W/m^2^]; ERF_htaL_ is the radiant heat transfer area combined with the effective radiation field for long-wave radiation in outdoor space [W/m^2^]; ERF_htaS_ is the radiant heat transfer area combined with the effective radiation field for short-wave solar radiation in outdoor space [W/m^2^]; ECF_hta_ is the heat transfer area combined effective conduction field [W/m^2^]; EHF_ETFe_ is the effective humid field at enhanced conduction-corrected modified effective temperature [W/m^2^]; *h*_rL_ is the radiant heat transfer coefficient for long-wave radiation in outdoor space [W/m^2 ^K]; fcl is the effective surface area factor of clothing [—]; *f*_conv_ is the convective heat transfer area factor [—]; *f*_cond_ is the conductive heat transfer area factor [—]; *f*_rad_ is the radiant heat transfer area factor [—]; Fcl is the thermal efficiency factor of clothing in the exposed airflow area [—]; Fcld is the thermal efficiency factor of clothing in the heat conduction area [—]; Fclo is the thermal efficiency factor of clothing under standard conditions [—]; Fpcl is the permeation efficiency factor of clothing [—]; *h*_*c*_ is the convective heat transfer coefficient [W/m^2 ^K]; *h*_*d*_ is the resultant heat conductance [W/m^2 ^K]; *h*_fL_ is the sensible heat transfer coefficient in outdoor space [W/m^2 ^K]; *h*_*o*_ is the convective heat transfer coefficient under standard conditions [W/m^2 ^K]; *L*: Lewis relation coefficient [K/kPa]; *p*_*a*_ is the water vapour pressure at outdoor air temperature [kPa]; *p*_ETFe_* is the saturated water vapour pressure at enhanced conduction-corrected modified effective temperature [kPa]; *R*_*S*_ is the short-wave solar radiation heat gain in human body [W/m^2^]; *T*_*s*_ is the convection-corrected mean skin temperature [K]; *T*_*f*_ is the surface temperature of the contacted material [K]; *T*_rL_ is the mean radiant temperature for long-wave radiation in outdoor space [K]; and *w* is the skin wetness [—].

### 2.2. Measurement Procedure

The measurements were largely carried out in winter, from January to March. Similarly to the measurement technique Kurazumi et al. [18] used in summer, field observations were carried out on foot. As a trolley was used to transport the thermal environment measuring instruments, the movement speed was slower than walking speed at around 0.7 m/s. The observation points were drawn at random and the route to the points was not fixed. To reduce the burden on subjects, the experiment was concluded two hours after commencement of the mobile observations on foot. Morning measurements were carried out from around 10:00 to 13:00, and afternoon measurements were carried out from around 13:00 to 16:00. 

The thermal environment of winter outdoor spaces can be harsh to the extent that the body temperature drops to the zone of body cooling. Accordingly, one must avoid extended periods in outdoor spaces where strong winds prevail in a low-temperature environment. Consequently, because of the subjects' standing position and the response time of the Assmann ventilated psychrometer, the actual measurement of the human body response and thermal environment in the mobile observations was performed after the observation device had been set up and left for five minutes. Naturally, it can be conjectured that the human body response will differ the longer the exposure time of the subjects, and the experimental period was determined with consideration for the safety of the subjects. Differently from indoor space, it is difficult to spend extended periods in an outdoor thermal environment as it would be uncomfortable because of behavioural thermoregulation by means of environmental refuge behaviour. 

Adaptation to the thermal environment according to the influence of thermal environment history is apparent, but a research method that removes the influence of environment history to the greatest possible extent was used in this research. Subjects moved on foot to the observation point after sitting and being at rest for 60 minutes or more in an indoor air-conditioned space at 22°C room temperature and 40% humidity in order to suppress the environment history. While they were seated and at rest, the subjects were informed that psychological reporting involves a thermally specific sense of thermal sensation and thermal comfort [9], that they would be asked to report an average sense during the period of exposure, and that intake and excretion of liquids were prohibited until the conclusion of the experiment. The migration speed of the subjects was around 0.7 m/s as detailed above because of the movement of the trolley in which the research staff transported the measurement instruments. 

After arriving at each measurement point, the subjects waited in a standing posture for five minutes while the test staff set up the measurement instruments for the thermal environment and preparations for measurement were concluded. Thereafter, the subjects were exposed to the thermal environment in a standing posture for five minutes, as shown above. The subjects were positioned around 1.5 m away from the centre of the thermal environment measurement instruments in a location where they did not obstruct the sunlight and they surrounded the thermal environment measurement instruments. In the case of observation points 4 and 10, which were located on pavements, the subjects were positioned directly facing the road with the thermal environment measurement instruments as the focus because of space considerations. As the subject of the research was the environment surrounding the observation stations, the point of gaze of the subjects was free and unfixed. After five minutes' exposure, the subjects reported the average thermal sensation and the average thermal comfort for the whole body that they experienced while exposed at the observation point.

### 2.3. Outline of the Observation Points

With reference to the measurements Kurazumi et al. [18] carried out in summer, 15 observation points were selected after consideration of the ground surface: bare ground where the surface was gravel or soil; paved ground such as concrete, asphalt, or blocks; green areas covered in plants; and water surfaces. The observation points were also selected with regard to the sky factor and the presence of buildings, trees, and so forth and the proportion of the solid angle of components of greenery, water, and so forth comprising the solid angle of the total celestial sphere (hereafter, green cover ratio).  Table 1 shows a summary of the observation points. 

### 2.4. Subjects

The subjects were 20 healthy young females. Their age was 22.0 ± 2.1, their height was 157.5 ± 3.4 cm, and their weight was 50.4 ± 5.6 kg. With a BMI of 20.3 ± 2.0, they can be considered to be unremarkable test subjects. In accordance with the Helsinki Declaration [27], the details of the experiment were explained to the subjects in advance, and their consent to participation in the experiment was obtained. 

### 2.5. Measurement Items

In order to maintain consistency with the measurements Kurazumi et al. [18] carried out in summer, the same measurement items as for the summer measurements were taken. Air temperature and humidity, air velocity, short-wave solar radiation, long-wave thermal radiation, ground surface temperature, and water surface temperature were measured as thermal environment conditions. The air temperature and humidity were measured at a height of 0.9 m above the ground by means of an Assmann ventilated psychrometer. The average air velocity was measured for five minutes at 1.2 m above the ground by a nondirectional hot-bulb air velocity sensor (Kanomax Japan, Inc.: 6533, measurement range 0.05~30.0 m/s). Concerning the short-wave solar radiation in the regions from the visible to the near- and-mid-infrared and the long-wave thermal radiation of the terrestrial radiation in the far infrared region, radiation quantities in both the downwards and upwards directions were measured at a height of 0.9 m above the ground by long- and short-wave radiometers (EKO Instruments: MR-50, sensitivity 7 *μ*V/Wm^−2^, short-wave range 305~2800 nm, long-wave range 5000~50000 nm). Ground surface temperature in the vicinity of the human body was measured by a radiation thermometer (Konica Minolta: HT-10D, measurement wave 8~14 *μ*m, measurement angle 1.4~2°, emissivity measurement range 0.10~1.00). The sky factor was measured by a photograph of the sky taken 1.2 m above the ground at the observation point with a fisheye lens with an orthographical projection format (Nikon: OP Fisheye Nikkor 10 mm f/5.6) and a 35 mm digital SLR camera. The proportion of the solid angle of components of greenery, water, and so forth comprising the solid angle of the total celestial sphere was measured by a photograph of the sky taken 1.2 m above the ground at the observation point with a fisheye lens with an equisolid angle projection format (Olympus: Fisheye Zuiko 8 mm f/2.8) and a 35 mm digital SLR camera. The albedo, sky temperature, and ground surface temperature were calculated from each directional component of the short-wave solar radiation and the long-wave thermal radiation. Furthermore, since the ground surface temperature in the vicinity of the human body is essential for the calculation of transmission heat quantity, values measured by radiation thermometer were used. Also, the values calculated by long- and short-wave solar radiometers were used as the average surface temperature and average sky temperature for the calculation of long-wave thermal radiation. 

With regard to the physiological conditions of the human body, the skin temperature of the part exposed to the air velocity was measured by a thermistor thermometer (Nikkiso-Therm, N542R and ITP8391, measurement range −50~230°C, resolution 0.01°C), and the skin temperature of contact parts was measured by a heat flux temperature sensor (Captec Enterprise, HF series, 0.4 mm thick, sensitivity 1.69–2.10 mV/(W/m^2^), response time 200 ms, T type thermocouple measurement range −50~230°C, one side painted black). The temperature of skin exposed to the air velocity was measured at the seven positions of the head, trunk, arm, hand, thigh, lower leg, and foot. The sole of the foot was measured for the temperature of the contact part skin. The subjects chose clothing suitable for the weather on the measurement day. The clothing quantity of the subjects was sought by the clo value by layering the clothing reported by the subjects [28]. 

Psychological response was measured after subjects had stayed at the observation point for five minutes by rating the whole-body thermal sensation (cold-hot) and the whole-body thermal comfort (comfortable-uncomfortable) on a linear scale [29, 30]. Only a direction was given for each scale, and reported values were rated from zero to 100. 

ETFe is an outdoor thermal environment evaluation index based on the heat balance of the human body. Accordingly, a weighting factor that takes into account the convection area of heat transfer surface was used for the calculation of the average skin temperature used to calculate the heat balance of the human body [31]. Then, the average skin temperature used for the physiological response of the human body was calculated by means of a weighting factor that takes into account heat conduction [32]. The values of Kurazumi et al. [33] were used for the convective heat transfer area factor, the radiant heat transfer area factor and the conduction heat transfer area factor, for the human body. The value of Miyamoto et al. [34] was used for the projection ratio of the human body. The values of Kuwabara et al. [35] were used for the radiant heat transmissibility and convective heat transmissibility of the human body. Hendler et al.'s [36] value of 0.98 found for the reflectance of skin in electromagnetic waves of wavelength 3 *μ*m or more was used for the emissivity of the human body. Hendler et al.'s [36] and Elam et al.'s [37] value of 0.70 found for the reflectance of skin in electromagnetic waves of wavelength 3 *μ*m or less was used for the solar radiation absorption factor of the human body. The heat transfer of short-wave solar radiation is affected by the solar radiation absorption factor. According to VDI3787-2 [38], the absorptivity of a clothed human body is 0.7. Watanabe et al. [39] showed, however, that the absorptivity of a human body wearing black clothes is 0.76 and that of one wearing white clothes is 0.38. They also considered the solar radiation absorption factor for other combinations of clothing or ordinary clothes to be within the range of the absorptivity of a human body wearing black clothes and that of one wearing white clothes. In this research, the absorptivity of the human body was based on the naked body and taken to be 0.7. With regard to skin wetness, values calculated by the Two-Node model [40] were used because it was difficult to find the perspiration quantity. ETFe was calculated from weather observation values, the skin temperature of the human body, and clothing quantity. 

## 3. Weather Synopsis

The measurement data for the observation points are shown in Table 2. The short-wave solar radiation downwards differed greatly between sun and shade. Cases where the ground surface temperature was under 0°C are also shown. Although there is also an effect of air temperature, the effect of heating by short-wave solar radiation and cooling by radiation from the ground surface can be identified. Although the contact area between the human body in a standing position and the ground surface is small, the heat acquisition of the human body by heat conduction is conjectured to have a strong effect on the contact skin temperature. Air velocity was comparatively gentle at about 3 m/s throughout all observations. Accordingly, the influence that the difference in convection heat exchange has on sensational and physiological temperature is conjectured to be weak. 

## 4. Relationship between the Components of ETFe and Thermal Sensation Vote

**Figure 1:**
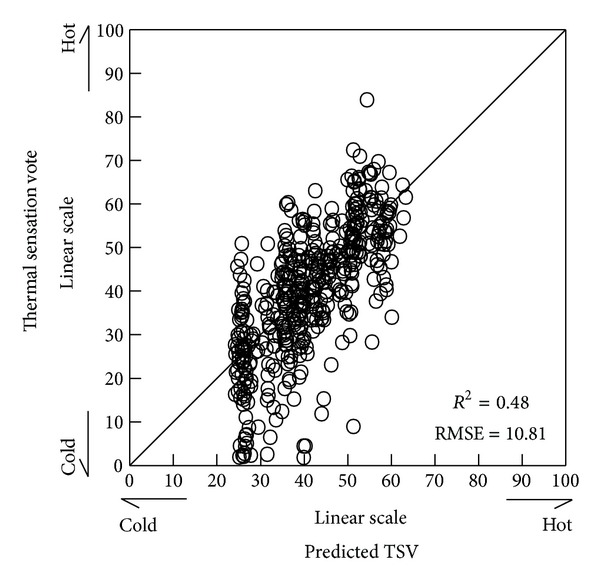
Relation between predicted thermal sensation vote and thermal sensation vote.

In order to predict the thermal sensation votes for the human body, multiple linear regression analysis was performed with air temperature *T*_*a*_ and air velocity, which are components of ETFe  TVF_hta_, long-wave thermal radiation ERF_htaL_, heat conduction ECF_hta_, humidity EHF_ETFe_, and short-wave solar radiation ERF_htaS_ as explanatory variables. The results are shown in Table 3. The relationship between the measured thermal sensation votes and values predicted for the thermal sensation votes by the multiple regression equation is shown in Figure 1. 

Being negative, the partial regression constant for air velocity  TVF_hta_ had a different symbol from other environmental factors. The convection heat exchange because of air velocity  TVF_hta_ functions as a heat loss for the human body, but heat exchange owed to other environmental factors functions as a heat gain for the human body. Accordingly, air velocity controls the direction of increase or decrease of thermal sensation vote owed to other environmental factors, and so all environmental factors were employed as explanatory variables in the multiple regression formula. 

A laboratory experiment is an environment in which it is easy to control environmental stimuli and human body conditions. Office spaces permit a wider range of thermal environments than laboratory space controlled by a supervisor. In turn, the living space in which a broader range of behaviour is available to the individual permits a wider range of thermal environment than does the office space. Finally, outdoor space which may be thermally uncomfortable but in which one's selection of location or point of attention may be changed at will permits a broader range of thermal environment than living space. That is, there is a significant dispersion in the psychological response of the human body in outdoor space. The coefficient of determination for the multiple regression formula depends on the experimental judgement of the analyst. The coefficient of determination for the multiple regression formula found in this study was 0.48, and the multiple regression formula was considered to be quite good. Furthermore, *P* < 0.01 as a result of analysis of variance on the multiple regression formula proved that the formula was valid.

As a standard for selection of explanatory variables in order to derive multiple linear regression analysis results that are more useful in practice, the objective significance probability was sometimes made 20–30%. Given that the psychological quantity for the human body in an outdoor environment differed from the case of an indoor environment and the results showed a lot of noise and dispersion, investigations were carried out with a significance probability of 25%. As a result of *t*-testing the components of ETFe, it was found that the thermal sensation vote for outdoors in winter is strongly influenced by and varies according to air temperature *T*_*a*_, long-wave thermal radiation ERF_htaL_, and short-wave solar radiation ERF_htaS_. As stated in the previous results previous, the air velocity was comparatively gentle at about 3 m/s throughout all observations, and it is possible that the difference in convection heat exchange had little influence on sensational and physiological temperature. Also, in winter, footwear with thick soles and excellent heat insulation is generally worn in order to protect against the cold. Accordingly, it can be said that the heat acquisition of the human body because of heat conduction did not influence thermal sensation votes even though the ground surface temperature was low because of the effect of insulation. 

Dealing with the relationship between outdoor environmental elements and thermal sensation votes, Givoni et al. [20], Oliveira and Andrade [21], Eliasson et al. [22], Nikolopoulou and Steemers [5], Nikolopoulou and Lykoudis [7], and others considered that short-wave solar radiation, air velocity, and air temperature strongly influence thermal sensation votes. Kurazumi et al. [18] demonstrated that the summer outdoor environmental factors which influence the thermal sensation vote of the human body are heat conduction, humidity and short-wave solar radiation. Ishii et al. [23], who dealt with the relationship between outdoor environmental elements in winter and thermal sensation votes, considered air temperature, humidity, and short-wave solar radiation to have a strong influence on thermal sensation votes. In this research, the same trend is shown concerning air temperature and short-wave solar radiation; however, results different from those of Ishii et al. [23] are shown for other factors. 

## 5. Relationship between the Components of ETFe and Thermal Comfort Vote

**Figure 2:**
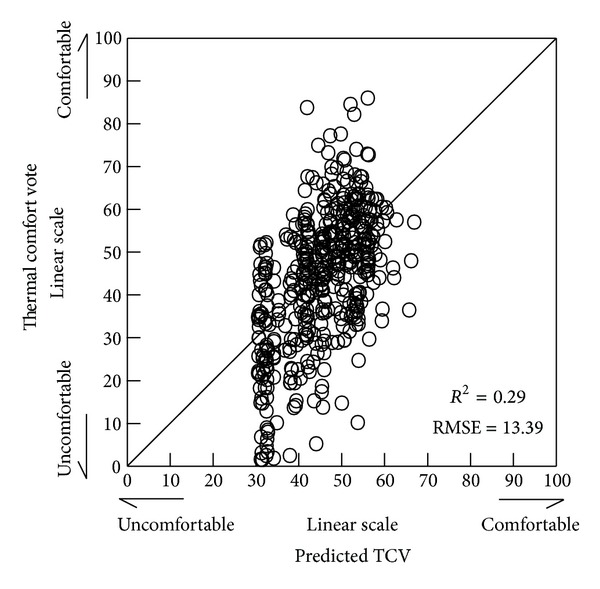
Relation between predicted thermal sensation vote and thermal comfort vote.

In order to predict the thermal comfort votes for the human body, multiple linear regression analysis was performed with air temperature *T*_*a*_ and air velocity, which are components of ETFe  TVF_hta_, long-wave thermal radiation ERF_htaL_, heat conduction ECF_hta_, humidity EHF_ETFe_, and short-wave solar radiation ERF_htaS_ as explanatory variables. The results of the multiple linear regression analysis for the thermal comfort votes, which were the objective parameters, are shown in Table 4. The relationship between the measured thermal comfort votes and values predicted for the thermal comfort vote by the multiple regression equation is shown in Figure 2. 

Similarly to the relationship between the above components of ETFe and thermal sensation votes, the partial regression constant for air velocity  TVF_hta_ differed from other environmental factors inasmuch that it was negative. Air velocity  TVF_hta_ controls the direction of increase or decrease of thermal comfort vote, however, because of other environmental factors, and so all environmental factors were employed as explanatory variables in the multiple regression formula.

The coefficient of determination for the multiple regression formula was 0.29. Although the analysis of the multiple regression formula cannot be said to be accurate, the multiple regression formula was shown to be valid, with *P* < 0.01 resulting from analysis of variance on the multiple regression formula. The poor analytical accuracy may have been influenced by environmental factors other than thermal environment stimuli. In this research, the experiment was carried out after the subjects had been informed that comfort is specific to heat. It was, however, implied that there is a psychological tendency to perform a nonspecific and comprehensive evaluation of the entire space including visual and auditory stimuli, when one is outdoors [10–14]. 

As a result of *t*-testing the components of ETFe, it was shown that the thermal comfort vote for outdoors in winter is easily influenced by air temperature *T*_*a*_, humidity EHF_ETFe_, short-wave solar radiation ERF_htaS_, long-wave thermal radiation ERF_htaL_, and heat conduction ECF_hta_. Considering that the air velocity was comparatively gentle at about 3 m/s throughout all observations, it is conjectured that the thermal comfort vote for outdoors in winter is influenced by all environmental factors addressed in this research. Accordingly, it may be necessary to treat short-wave solar radiation and heat conduction in the thermal environment evaluation of an outdoor space. Ishii et al. [23] demonstrated that the winter outdoor environmental factor which influences the thermal comfort vote of the human body is short-wave solar radiation. Kurazumi et al. [18] demonstrated that the summer outdoor environmental factors which influence the thermal comfort vote of the human body are air velocity, heat conduction, and humidity. 

Although it is physically possible to handle environmental stimuli, it is difficult to handle the human body effect on the environmental stimuli independently. For example, in the case of a hot environment in summer where the temperature is higher than skin temperature, the heat balance of the human body dropping as the air velocity increases can promote a sense of discomfort. In addition, in the case of a cooling winter environment of a temperature lower than the air temperature because of radiative cooling, the heat balance of the human body as the air velocity increases can show a thermal stimulus close to the air temperature. 

In the above, the response of the thermal sense to the environmental stimuli is subsidiary, but when the rate at which the environment stimuli contribute to thermal sense is investigated, it is appropriate to consider the following. In the evaluation of a winter outdoor environment, it has been demonstrated that it is essential to incorporate short-wave solar radiation and heat conduction as evaluation factors in addition to air temperature and air velocity, humidity, and long-wave thermal radiation. Given the results of Ishii et al. [23] for air velocity measurement and winter outdoor environmental factors and those of Kurazumi et al. [18] for summer outdoor environmental factors, we have demonstrated that it is essential to include short-wave solar radiation and heat conduction in the evaluation of outdoor thermal environments. 

## 6. Conclusions 

An experiment on test subjects was performed to clarify the effect that the thermal environment stimuli in a winter outdoor space have on the human body, and the relationship between outdoor environmental factors and the psychological response of the human body was demonstrated. In addition to air temperature and humidity, air velocity, and long-wave thermal radiation, it was found that short-wave solar radiation and heat conduction are among the influential factors which affect the thermal sensation vote and thermal comfort vote of the human body in a winter outdoor thermal environment. The validity of using a thermal environment evaluation index that incorporates these environmental factors for an outdoor space was demonstrated. 

## Figures and Tables

**Table 1:** Summary of observation points.

Point	Survey site	Ground surface	Skywards surface	Surrounds north side	Surrounds east side	Surrounds south side	Surrounds west side	Sky factor	Green factor
1	University campus	Paving brick	Open	Open	Open	Building	Building	0.819	0.005
2	Building canyon	Concrete	Roof	Open	Open	Building	Building	0.323	0.004
3	Building side	Concrete	Open	Open	Open	Building	Building	0.443	0.000
4	Residential street	Asphalt	Open	Building	Open	Wall	Open	0.701	0.021
5	Community playground	Bare ground	Open	Tree	Tree	Tree	Tree	0.861	0.015
6	Park green area	Dead grass	Tree	Tree	Tree	Tree	Tree	0.766	0.361
7	Community park	Dead weed	Tree	Tree	Tree	Tree	Tree	0.632	0.300
8	Park road	Asphalt	Tree	Open	Tree	Open	Wall and tree	0.497	0.207
9	Parking	Asphalt and reservoir	Open	Open	Open	Open	Open	0.978	0.055
10	Road side	Paving brick	Tree	Tree	Open	Tree	Open	0.570	0.100
11	Temple precincts	Gravel	Open	Building	Open	Open	Slope	0.809	0.036
12	Promenade	Asphalt	Bamboo	Open	Bamboo	Open	Bamboo	0.447	0.269
13	Urban canyon	Paving tile	Roof	Open	Building	Open	Building	0.482	0.007
14	Promenade	Wooden deck	Open	Open	Building	Open	Open	0.734	0.033
15	University park	Brick, grass and pond	Open	Tree	Tree	Building	Tree	0.340	0.258

Green factor is green covering factor. Green covering factor is defined as the ratio of green, water surface solid angles to celestial globe solid angle.

**Table 2:** Results of field survey.

Date	Period	Survey site	Number of subjects	*T*_*a*_ (°C)	*T*_*f*_ (°C)	RH (%)	*V*_*a*_ (m/s)	*RS*_dwn_ (W/m^2^)	*RS*_up_ (W/m^2^)	*RL*_dwn_ (W/m^2^)	*RL*_up_ (W/m^2^)
27 Jan.	13:30–14:20	1, 15	12	4.8–5.3	−0.5–3.6	48.4–52.4	1.1–3.7	60.9–67.1	4.7–7.8	273.4–310.0	334.8–349.2
31 Jan.	13:50–15:00	2, 3, 4, 5, 6	12	2.2–4.5	−1.3–3.4	50.3–62.4	0.6–1.6	5.0–149.5	–14.8–37.4	311.0–331.6	340.0–379.6
1 Feb.	13:35–15:40	1, 11, 12, 13, 14, 15	9	6.0–15.3	1.9–21.6	29.0–59.4	0.6–2.9	15.1–497.7	–9.9–64.4	207.8–273.7	274.7–337.3
2 Feb.	14:00–15:40	1, 7, 8, 9, 10, 15	11	8.8–11.0	6.7–13.3	18.6–25.1	0.4–2.9	67.1–255.8	1.4–44.2	305.5–346.7	365.4–397.3
18 Feb.	10:45–12:15	10, 11, 12, 13, 14	8	10.3–12.9	8.6–21.1	35.2–39.6	1.2–2.3	63.8–711.4	3.4–104.0	304.0–341.0	376.5–411.6
21 Feb.	10:35–15:30	2, 3, 4, 5, 6, 7, 8, 9, 10, 11, 12, 13, 14	6	10.1–15.7	6.2–36.2	19.5–36.2	0.3–2.3	7.4–746.8	−9.6–145.4	275.3–482.0	378.3–450.7
23 Feb.	10:40–12:20	7, 8, 9, 10, 13, 14	4	13.5–18.5	9.5–25.7	22.3–36.5	0.9–2.4	66.6–688.4	14.0–140.7	280.9–339.7	383.8–457.6
3 Mar.	10:25–11:30	2, 3, 4, 5, 6	3	5.1–7.5	1.9–18.8	22.8–28.7	0.8–6.6	30.8–790.0	−6.4–143.6	294.2–331.7	354.0–408.2
11 Mar.	14:10–14:40	2	4	8.3	7.5	42.5	0.6	64.3	5.9	350.1	386.6
14 Mar.	10:50–13:40	2, 3, 4, 5, 6, 7, 8, 9, 10, 13, 14	3	14.1–21.3	1.9–28.5	25.3–40.3	0.3–1.9	87.0–603.6	16.0–164.4	308.0–368.0	383.8–427.9
17 Mar.	10:00–11:20	2, 3, 4, 5, 6	3	4.6–6.9	3.8–23.9	25.3–32.0	0.9–2.1	33.4–646.2	44.1–128.5	296.1–322.3	365.9–416.9
18 Mar.	10:40–11:45	2, 3, 4, 5, 6	5	6.5–8.0	4.3–23.1	23.5–25.9	0.5–2.1	72.7–794.0	14.4–181.6	266.5–319.8	364.2–406.3
22 Mar.	11:40–12:00	4	3	16.0	25.4	41.7	2.1	733.4	49.3	335.6	420.7
25 Mar.	10:50–12:55	7, 8, 9, 11, 12, 13, 14	3	10.6–13.8	11.5–33.4	26.6–29.1	0.9–24	88.7–830.9	10.8–170.2	268.2–343.2	386.4–472.0
28 Mar.	11:20–12:05	7, 8, 9	3	12.0–12.7	20.3–38.2	9.9–11.4	1.1–1.9	552.6–933.3	44.5–183.1	258.0–340.6	419.1–461.7
29 Mar.	9:30–10:10	11, 12	8	13.2–13.9	11.8–22.3	24.9–26.0	0.9–1.5	329.0–698.1	36.3–82.0	221.5–247.8	331.2–360.5

*T*_*a*_ is the range of air temperature. *T*_*f*_ is the range of ground surface temperature in the vicinity of the human body. RH is the range of relative humidty. *V*_*a*_ is the range of air velocity. *RS*_dwn_ is the range of downward short-wave solar radiation. *RS*_up_ is the range of upward short-wave solar radiation. *RL*_dwn_ is the range of downward long-wave radiation. *RL*_up_ the is range of upward long-wave radiation.

**Table 3:** Results of multiple linear regression analysis in case of thermal sensation vote.

Explanatory variable	Partial regression coefficient	Standard error	*t* score	*P* value
Interception	22.73	5.31	4.28	0.00
T_a_	1.74	0.21	8.14	0.00
TVF_hta_/*h*_fL_	−0.27	0.30	−0.91	0.36
ERF_htaL_/*h*_fL_	0.89	0.49	1.82	0.07
ECF_hta_/*h*_fL_	68.43	73.74	0.93	0.35
EHF_ETFe_/*h*_fL_	86.07	117.94	0.73	0.47
ERF_htaS_/*h*_fL_	0.70	0.17	4.15	0.00

Response variable is thermal sensation vote.

**Table 4:** Results of multiple linear regression analysis in case of thermal comfort vote.

Explanatory variable	Partial regression coefficient	Standard error	*t* score	*P* value
Interception	27.19	6.57	4.14	0.00
T_a_	1.86	0.26	7.06	0.00
TVF_hta_/*h*_fL_	−0.16	0.37	−0.43	0.67
ERF_htaL_/*h*_fL_	0.85	0.61	1.40	0.16
ECF_hta_/*h*_fL_	113.63	91.26	1.25	0.21
EHF_ETFe_/*h*_fL_	367.66	145.98	2.52	0.01
ERF_htaS_/*h*_fL_	0.38	0.21	1.82	0.07

Response variable is thermal comfort vote.
